# Skeletal muscle gauge as a prognostic factor in patients with colorectal cancer

**DOI:** 10.1002/cam4.4354

**Published:** 2021-10-13

**Authors:** In Kyu Park, Song Soo Yang, Eric Chung, Eun‐Suk Cho, Hye Sun Lee, Su‐Jin Shin, Yeong Cheol Im, Eun Jung Park, Seung Hyuk Baik, Kang Young Lee, Jeonghyun Kang

**Affiliations:** ^1^ Department of Surgery Ulsan University Hospital, University of Ulsan College of Medicine Ulsan Republic of Korea; ^2^ Department of Anesthesiology Indiana University Indianapolis Indiana USA; ^3^ Department of Radiology, Gangnam Severance Hospital Yonsei University College of Medicine Seoul Republic of Korea; ^4^ Biostatistics Collaboration Unit Yonsei University College of Medicine Seoul Republic of Korea; ^5^ Department of Pathology, Gangnam Severance Hospital Yonsei University College of Medicine Seoul Republic of Korea; ^6^ Department of Surgery, Gangnam Severance Hospital Yonsei University College of Medicine Seoul Republic of Korea; ^7^ Department of Surgery, Severance Hospital Yonsei University College of Medicine Seoul Republic of Korea

**Keywords:** colorectal cancer, myosteatosis, sarcopenia, skeletal muscle gauge, skeletal muscle index, skeletal muscle radiodensity

## Abstract

**Background:**

Although skeletal muscle index (SMI) and radiodensity (SMD) are well‐known prognostic factors, the clinical impact of the integrated measure, known as skeletal muscle gauge (SMG), has been limited in patients with colorectal cancer (CRC).

**Patients and Methods:**

A total of 727 and 268 patients with CRC at two tertiary centers were included and allocated into the training and test sets, respectively. Preoperative slice computed tomography images of the third lumbar area were evaluated for SMI and SMD. SMG was calculated as SMI × SMD and expressed as an arbitrary unit (AU). The optimal cutoff SMG value was determined to maximize the overall survival (OS) difference between the groups with respect to sex in the training set. The multivariate Cox proportional hazard model evaluated the association of its clinical significance.

**Results:**

With regard to SMG, 1640 and 1523 AU were identified as cutoff values for males and females, respectively. The patients with low SMG values showed significantly worse 5‐year OS than those with high SMG values in the two datasets (both *p *< 0.001). In the multivariate analysis, low SMG was identified as an independent poor prognostic factor of OS in the training set (hazard ratio 2.18, 95% confidence interval 1.43–3.32, *p *< 0.001) and test set (hazard ratio 1.79, 95% confidence interval 1.07–3.00, *p *= 0.025), whereas SMI and SMD were not.

**Conclusion:**

SMG acts synergistically to improve its prognostic predictive accuracy as compared with SMI or SMD alone in patients with CRC. Additional research is warranted to define its significance in different ethnic groups.

## Graphical abstract

Skeletal muscle gauge acts synergistically to improve its prognostic predictive accuracy as compared with skeletal muscle index or skeletal muscle radiodensity alone in patients with colorectal cancer. Additional research is warranted to define its significance in different ethnic groups.

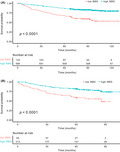

## 1 INTRODUCTION

The terminology of sarcopenia was initially used to describe age‐associated loss of muscle mass in elderly persons.^1^ Sarcopenia has been investigated even in the field of oncology.^2^, ^3^, ^4^, ^5^, ^6^ Low skeletal muscle index (SMI) has been suggested to predict higher postoperative complication rates or poor survival in patients with cancer.^7^, ^8^ With the wide application of computed tomography (CT) in measuring skeletal muscle characteristics, an increasing body of research has been focused on the clinical significance of skeletal muscle radiodensity (SMD), which can be referred to as an evaluation of muscle quality. Recent meta‐analyses reported that myosteatosis could be used as a significant predictor of survival in various types of cancers, including CRC.^9^, ^10^

Furthermore, the impact of skeletal muscle gauge (SMG), which was defined as the product of SMI and SMD, has been recently evaluated.^11^, ^12^, ^13^, ^14^ Shachar et al. reported that low SMG was associated with grade 3 or 4 toxicity and more hospitalizations in patients with metastatic breast cancer.^11^ It was reported that SMG loss more than 5% per 210 days during staging surgery and adjuvant chemoradiotherapy was independently associated with survival in patients with endometrial cancer.^14^ However, the role of SMG has been evaluated limitedly with a relatively small number of female patients.

The clinical significance of SMI and SMD has been investigated broadly in patients with CRC.^3^, ^4^, ^5^, ^6^, ^15^, ^16^, ^17^, ^18^, ^19^ Some studies reported that patients with both sarcopenia and myosteatosis showed worse survival than those who only had sarcopenia or myosteatosis.^16^, ^19^ These observations show the possibility that combining SMI and SMD will have a synergistic effect in predicting patient outcomes and this concept has already been proposed previously.^20^ Interest in the prognostic role of SMG is growing, and a recent study showed that patients with low SMG and high total adiposity tissue area had inferior survival in patients with metastatic melanoma treated with immune check point inhibitors.^21^ Although SMG has been suggested as a combination of the two skeletal muscle‐related factors, evaluation of the potential prognostic effect of SMG has been limited in patients with CRC.

Thus, the aim of this study was to investigate the prognostic significance of SMG and to confirm the clinical impact of newly developed cutoff values using an independent dataset in patients with CRC.

## 2 MATERIALS AND METHODS

### 2.1 Patients

This retrospective study was conducted in patients diagnosed as having CRC and treated surgically in two tertiary institutions, namely Gangnam Severance Hospital, Yonsei University College of Medicine (training set); and Ulsan University Hospital, University of Ulsan College of Medicine (test set). The medical records of patients who were diagnosed as having stage I–IV CRC and underwent curative intent surgery between January 2009 and April 2014 in the training set and between November 2011 and June 2013 in the test set were retrospectively analyzed.

The following patients were eligible for study inclusion: (1) diagnosed as having stage I–IV CRC; (2) underwent a routine abdominal−pelvic computed tomography within 60 days before surgery; and (3) underwent a curative intent resection. The exclusion criteria included the following: (1) incomplete follow‐up or clinical data; (2) unavailable skeletal muscle index or radiodensity; (3) underwent preoperative chemoradiotherapy; (4) had double primary cancers; (5) had Crohn's disease, familiar adenomatous polyposis, or hereditary non‐polyposis syndrome; (6) underwent emergency operations; and (7) had appendiceal cancer or recurrent colorectal cancers. As the mean SMD value might be different between the non‐contrast and contrast‐enhanced CT images,^22^ contrast‐enhanced scans such as those taken in the arterial or venous phase were used for the analysis. Thus, patients who only had non‐contrast CT images were also excluded from our analysis (Figure S1).

### 2.2 Measurements of SMI, SMD using CT images, and calculation of SMG

CT images of the skeletal muscle were obtained at the level of the third lumbar vertebra. For measuring skeletal muscle area (SMA), the cross‐sectional L3 CT slice was entered into the in‐house open‐source software named BMI_CT, which is available online (https://sourceforge.net/projects/muscle‐fat‐area‐measurement).^23^ In addition, the SMD was measured using an open‐source software named as 3DSlicer (https://www.slicer.org/).^24^ The intra‐class correlation coefficients of SMI and SMD as determined by two investigators using these two software were 0.97 (range 0.95–0.99) and 0.99 (0.97–0.99), respectively, in our previous study.^6^ SMA (cm^2^) was measured using Hounsfield units (HU) ranging from −29 to +150. SMA was normalized for height to derive the SMI (cm^2^/m^2^). The SMD was the mean HU of the SMA. The method was described in detail in our previous work.^6^

With regard to SMI and SMD, the patients were classified as having a sarcopenic status using the sex‐specific cutoff values suggested by Martin and colleagues (Table S1).^2^ SMG was calculated by multiplying the SMI and the SMD, as suggested by Weinberg and colleagues.^13^ For simplicity, instead of using (cm^2^×HU/m^2^) as the SMG unit, an arbitrary unit (AU) was used, as in other studies.

Given that the SMG may vary greatly with cancer types, local regions, and/or ethnicities, we needed to define a new cutoff value. The optimum cutoff SMG value was selected on the basis of the association with overall survival (OS) of patients in the training set using X‐tile Version 3.6.1 software (Yale University School of Medicine).^25^ The basic principle of determining the optimal cutoff value was to find the value that produced the largest *χ* ^2^ value in the Mantel–Cox test. This cutoff SMG value derived from the training set was applied to the test set to examine the potential association of SMG with OS. The patients were divided into low and high SMG subgroups on the basis of this value.

### 2.3 Follow‐up

The patients were followed up regularly according to each hospital's policy. The patients visited the hospital every 3 or 6 months for the first 3 years and every 6 months in the subsequent years. The patients received carcinoembryonic antigens at each visit. Abdominopelvic and/or chest CT scans were performed every 6–12 months after surgery. Pelvic magnetic resonance imaging, ^18^F‐fluorodeoxyglucose positron‐emission tomography, or colonoscopy were performed in accordance with the physician's decision. All these examinations, however, were modified depending on the staging and patient compliance. The patients were followed up until October 2019 (training set), August 2020 (test set), or death of the patient. The median follow‐up period was 89 months (interquartile range [IQR] 70–102 months) in the training set and 60 months (IQR 42–82 months) in the test set.

### 2.4 Statistical analysis

The distributions of clinical characteristics were compared using the chi‐squared test for categorical variables and the Wilcoxon rank sum test for all continuous variables.

OS was defined as the time from the date of surgery to the date of death from any cause or date of last follow‐up. OS was estimated using the Kaplan–Meier method and compared between the groups using the log‐rank test. The Cox proportional hazard model was used to analyze the hazard ratios (HRs) and 95% confidence intervals (CIs) for the factors associated with OS. Among the factors related to OS, only the variables found to be significant (*p *< 0.05) in univariate Cox regression analysis were included in the multivariate analysis.

Harrell's concordance index (C‐index) was used to confirm the predictive power of SMG for OS. First, we compared the C‐index between SMG and stage. In addition, we compared a model using stage and another model combining stage plus SMG to evaluate the incremental benefit of SMG for the prediction of prognosis.

A two‐sided *p* value <0.05 was considered significant. All statistical analyses were performed using R version 3.6.3 (R‐project, Institute for Statistics and Mathematics).

## 3 RESULTS

A total of 727 and 268 patients in the training and test sets were included in this study, respectively. The median (IQR) ages (years) were 63 (55–71) and 61 (54–70), respectively. In the training and test sets, 430 (59.1%) and 158 patients (58.9%) were men and 161 (31.2%) and 68 patients (25.3%) died during the study period, respectively.

### 3.1 Distribution of skeletal muscle‐related parameters in the training and test sets

The median SMI, SMD, and SMG were 52.6, 45.2, and 2356, respectively, in male patients and 43.2, 41.7, and 1769, respectively, in female patients (all *p *< 0.001) in the training set and 54.0, 46.9, and 2447, respectively, in male patients and 44, 40.4, and 1752, respectively, in female patients (all *p *< 0.001) in the test set (Table 1, Figure S2).

**TABLE 1:** Comparison of skeletal muscle index, skeletal muscle radiodensity, and skeletal muscle gauge according to male and female in the training and test sets

	Training set			Test set		
	Male (*n* = 430)	Female (*n* = 297)	*p*	Male (*n* = 158)	Female (*n* = 110)	*p*
SMI (median, IQR)	52.6 (46.5–57.7)	43.2 (39.1–47.8)	<0.001	54.0 (47.1–58)	44 (40.1–14.6)	<0.001
SMD (median, IQR)	45.2 (40.2–49.7)	41.7 (35.9–47)	<0.001	46.9 (39.6–54.2)	40.4 (29.6–47.2)	<0.001
SMG (median, IQR)	2356 (1950–2723)	1769 (1505–2021)	<0.001	2447 (2030–2916)	1752 (1338–2088)	<0.001

Abbreviations: IQR, interquartile range; SMD, skeletal muscle radiodensity;SMG, skeletal muscle gauge; SMI, skeletal muscle index.

### 3.2 Defining the cutoff SMG value in the training set

We defined sex‐specific cutoff values due to baseline differences in SMG according to sex in the training set. The cutoff SMG values were 1632 and 1523 AU in the male and female patients, respectively (Figure S3). A total of 132 (18.1%) and 55 patients (20.5%) were allocated into the low SMG groups in the training and test sets, respectively.

### 3.3 Comparison of patient characteristics according to low and high SMG values

Significant differences in sex, age, American Society of Anesthesiologists (ASA) grade, and receipt of chemotherapy and in sex, age, and stage were found between the low and high SMG groups in the training and test sets, respectively. SMI and SMD were significantly higher in the high SMG group in both sets (Table 2).

**TABLE 2:** Patient characteristics according to low and high skeletal muscle gauge in the training and test sets

	Training set (*n* = 727)			Test set (*n* = 268)		
	low SMG (*n* = 132) *N* (%)	high SMG (*n* = 595) *N* (%)	*p*	low SMG (*n* = 55) *N* (%)	high SMG (*n* = 213) *N* (%)	*p*
Sex						
Female	77 (58.3)	220 (37)	<0.001	40 (72.7)	70 (32.9)	<0.001
Male	55 (41.7)	375 (63)		15 (27.3)	143 (67.1)	
Age (years)						
<60	23 (17.4)	247 (41.5)	<0.001	12 (21.8)	107 (50.2)	<0.001
≥60	109 (82.6)	348 (58.5)		43 (78.2)	106 (49.8)	
ASA classification						
I	52 (39.4)	298 (50.1)	0.006	12 (21.8)	67 (31.5)	0.236
II	51 (38.6)	225 (37.8)		41 (74.5)	143 (67.1)	
III	29 (22)	72 (12.1)		2 (3.6)	3 (1.4)	
BMI (kg/m^2^)						
<25	94 (71.2)	418 (70.3)	0.910	34 (61.8)	145 (68.1)	0.473
≥25	38 (28.8)	177 (29.7)		21 (38.2)	68 (31.9)	
CEA (ng/ml)						
<5	77 (58.3)	393 (66.1)	0.072	33 (60)	154 (72.3)	0.206
≥5	51 (38.6)	172 (28.9)		18 (32.7)	49 (23)	
No data	4 (3)	30 (5)		4 (7.3)	10 (4.7)	
Tumor location						
Colon	98 (74.2)	422 (70.9)	0.511	45 (81.8)	174 (81.7)	>0.99
Rectum	34 (25.8)	173 (29.1)		10 (18.2)	39 (18.3)	
Histologic grade						
G1 & G2	116 (87.9)	552 (92.8)	0.092	48 (87.3)	197 (92.5)	0.337
G3 & MC & SRC	16 (12.1)	43 (7.2)		7 (12.7)	16 (7.5)	
LVI						
Absent	94 (71.2)	440 (73.9)	0.318	31 (56.4)	128 (60.1)	0.728
Present	36 (27.3)	135 (22.7)		24 (43.6)	85 (39.9)	
No data	2 (1.5)	20 (3.4)				
Stage						
I & II	70 (53)	314 (52.8)	0.997	21 (38.2)	122 (57.3)	0.031
III	47 (35.6)	212 (35.6)		26 (47.3)	64 (30)	
IV	15 (11.4)	69 (11.6)		8 (14.5)	27 (12.7)	
Complications						
No	105 (79.5)	473 (79.5)	>0.99	35 (63.6)	150 (70.4)	0.420
Yes	27 (20.5)	122 (20.5)		20 (36.4)	63 (29.6)	
Chemotherapy						
No	76 (57.6)	264 (44.4)	0.008	33 (60)	108 (50.7)	0.280
Yes	56 (42.4)	331 (55.6)		22 (40)	105 (49.3)	
SMI						
Median (IQR)	40.7 (8.0)	49.8 (8.3)	<0.001	43.3 (6.2)	50.0 (8)	<0.001
SMD						
Median (IQR)	32.0 (6.6)	45.4 (5.9)	<0.001	29.1 (6.5)	47.1 (7.6)	<0.001

Abbreviations: ASA, American Society of Anesthesiologists; BMI, body mass index; CEA, carcinoembryonic antigen; LVI, lymphovascular invasion; MC, mucinous adenocarcinoma; SMD, skeletal muscle radiodensity; SMI, skeletal muscle index; SRC, signet‐ring cell.

### 3.4 Kaplan–Meier curves according to OS

**FIGURE 1:**
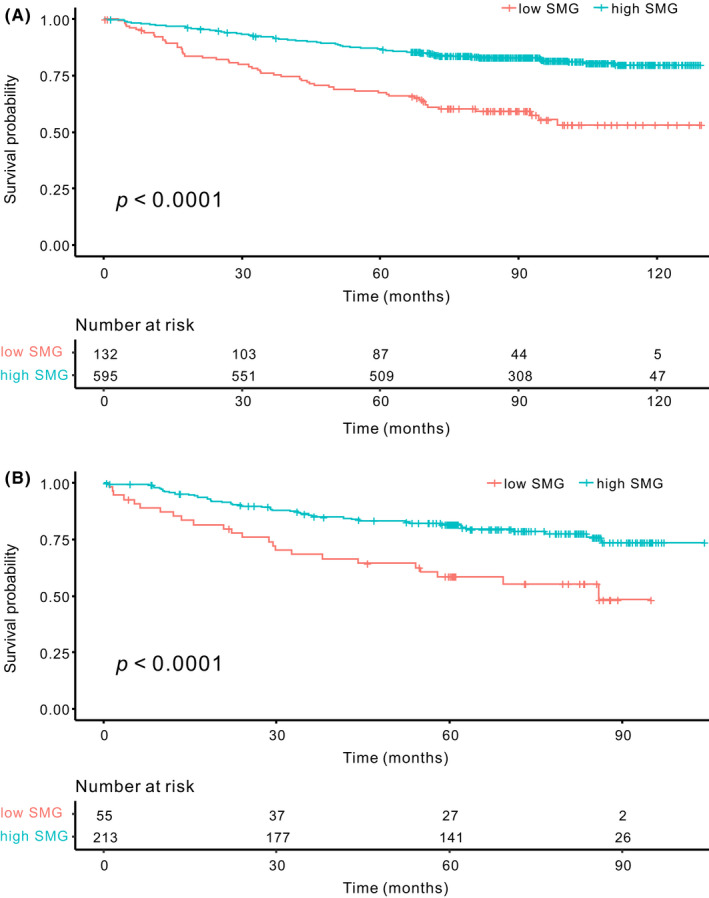
Kaplan–Meier survival curve. The overall survival of the high SMG group was significantly better than that of the low SMG group in both the training (A) and test sets (B) (both *p *< 0.0001).

The 5‐year OS rate was significantly lower in the low than in the high SMG group in both sets (both *p *< 0.0001; Figure 1). In the training set, the patients with low SMI and SMD showed worse OS than those with high SMI (*p *= 0.0037) and SMD (*p *< 0.0001). In the test set, we found no significant difference in OS between the patients with low and high SMI (*p *= 0.13), whereas patients with low SMD showed worse OS than those with high SMD (*p *= 0.029; Figure S4).

### 3.5 Univariate and multivariate analyses of OS

In the univariate Cox regression survival analysis in the training set, age (*p *= 0.009), body mass index (BMI; *p *= 0.027), preoperative carcinoembryonic antigen (CEA; *p *< 0.001), lymphovascular invasion (LVI; *p *< 0.001), stage (*p *< 0.001), complications (*p *< 0.001), chemotherapy (*p *< 0.001), SMI (*p *= 0.004), SMD (*p *< 0.001), and SMG (*p *<0.001) were significantly associated with OS. In the subsequent multivariate Cox regression analysis, age (HR, 95% CI: 1.57, 1.11–2.22, *p *= 0.009), BMI (0.64, 0.43–0.94, *p *= 0.024), CEA level (<5 vs. ≥5, 1.49, 1.06–2.09, *p *= 0.021), complications (1.7, 1.2–2.4, *p *= 0.002), LVI (absent vs. present, 1.56, 1.1–2.22, *p *= 0.012), TNM stage (I and II vs. III: 2.6, 1.68–4.01, *p *< 0.001; I and II vs. IV: 6.59, 4.13–10.5, *p *< 0.001), chemotherapy (0.41, 0.29–0.58, *p *< 0.001), and SMG (2.18, 1.43–3.32, *p *< 0.001) were identified to be independently associated with OS (Tables 3 and 4).

**TABLE 3:** Univariate analysis of factors associated with overall survival in the training and test sets

	Training set (*n* = 727)		Test set (*n* = 268)	
	HR (95% CI)	*p*	HR (95% CI)	*p*
Sex				
Female	1		1	
Male	1.35 (0.97–1.86)	0.071	1 (0.61–1.62)	0.997
Age (years)				
<60	1		1	
≥60	1.57 (1.11–2.22)	0.009	1.9 (1.16–3.24)	0.01
ASA classification				
I	1		1	
II	1.221 (0.87–1.7)	0.241	1.92 (1.04–3.53)	0.035
III	1.18 (0.73–1.91)	0.483	8.46 (2.39–29.9)	<0.001
BMI (kg/m^2^)				
<25	1		1	
≥25	0.65 (0.45–0.95)	0.027	0.71 (0.41–1.21)	0.2
CEA (ng/ml)				
<5	1		1	
≥5	2.57 (1.88–3.51)	<0.001	2.46 (1.49–4.07)	<0.001
No data	0.76 (0.27–2.08)	0.599	2.65 (1.11–6.3)	0.027
Tumor location				
Colon	1		1	
Rectum	0.94 (0.67–1.34)	0.769	1.44 (0.81–2.56)	0.209
Histologic grade				
G1 & G2	1		1	
G3 & MC & SRC	1.46 (0.88–2.41)	0.138	3.1 (1.65–5.82)	<0.001
LVI				
Absent	1		1	
Present	2.75 (2–3.79)	<0.001	2.03 (1.25–3.28)	0.003
No data	1.03 (0.37–2.81)	0.948		
Stage				
I & II	1		1	
III	2.12 (1.45–3.11)	<0.001	2 (1.1–3.62)	0.021
IV	8.48 (5.69–12.63)	<0.001	7.36 (4.05–13.37)	<0.001
Complications				
No	1		1	
Yes	1.89 (1.35–2.65)	<0.001	2.2 (1.37–3.55)	0.001
Chemotherapy				
No	1		1	
Yes	0.56 (0.41–0.77)	<0.001	0.36 (0.21–0.61)	<0.001
SMI				
High	1		1	
Low	1.61 (1.16–2.23)	0.004	1.48 (0.88–2.49)	0.13
SMD				
High	1		1	
Low	2.48 (1.81–3.45)	<0.001	1.69 (1.05–2.73)	0.03
SMG				
High	1		1	
Low	2.88 (2.08–4.00)	<0.001	2.49 (1.51–4.09)	<0.001

Abbreviations: ASA, American Society of Anesthesiologists; BMI, body mass index; CEA, carcinoembryonic antigen; CI, confidence interval; HR, hazard ratio; LVI, lymphovascular invasion; MC, mucinous adenocarcinoma; SMD, skeletal muscle radiodensity; SMG, skeletal muscle gauge; SMI, skeletal muscle index; SRC, signet‐ring cell.

**TABLE 4:** Multivariate analysis of factors associated with overall survival in the training and test sets

	Training set (*n* = 727)		Test set (*n* = 268)	
	HR (95% CI)	*p*	HR (95% CI)	*p*
Age (years)				
<60			1	
≥60			1.87 (1.02–3.41)	0.04
ASA classification				
I			1	
II			1.42 (0.71–2.82)	0.316
III			9.65 (2.37–39.26)	0.001
BMI (kg/m^2^)				
<25	1			
≥25	0.64 (0.43–0.94)	0.024		
CEA (ng/ml)				
<5	1			
≥5	1.49 (1.06–2.09)	0.021		
No data	0.86 (0.31–2.42)	0.788		
Complications				
No	1		1	
Yes	1.7 (1.2–2.4)	0.002	1.86 (1.11–3.1)	0.021
Histologic grade				
G1 & G2			1	
G3 & MC & SRC			3.45 (1.77–6.71)	<0.001
LVI				
Absent	1		1	
Present	1.56 (1.1–2.22)	0.012	1.78 (1.03–3.1)	0.035
No data	1.12 (0.4–3.1)	0.827		
Stage				
I & II	1		1	
III	2.6 (1.68–4.01)	<0.001	2.78 (1.4–5.51)	0.003
IV	6.59 (4.13–10.5)	<0.001	7.75 (3.79–15.85)	<0.001
Chemotherapy				
No	1		1	
Yes	0.41 (0.29–0.58)	<0.001	0.26 (0.14–0.47)	<0.001
SMD				
High	1			
Low	1.40 (0.94–2.08)	0.09		
SMG				
High	1		1	
Low	2.18 (1.43–3.32)	<0.001	1.79 (1.07–3.00)	0.025

Abbreviations: ASA, American Society of Anesthesiologists; BMI, body mass index; CEA, carcinoembryonic antigen; CI, confidence interval; HR, hazard ratio; LVI, lymphovascular invasion; MC, mucinous adenocarcinoma; SMD, skeletal muscle radiodensity;SMG: skeletal muscle gauge; SRC, signet‐ring cell.

In the test set, the univariate analysis revealed that age (*p *= 0.01), ASA grade (*p *< 0.001), CEA level (*p *< 0.001), histological grade (*p *< 0.001), LVI (*p *= 0.003), stage (*p *< 0.001), complications (*p *< 0.001), chemotherapy (*p *< 0.001), SMD (*p *= 0.03), and SMG (*p *< 0.001) were significantly associated with OS. The multivariate analysis revealed that age (HR, 95% CI: 1.87, 1.02–3.41, *p *= 0.04), ASA grade (I vs. III: 9.65, 2.37–39.26, *p *= 0.001), complications (1.86, 1.11–3.1, *p *= 0.021), histological grade (3.45, 1.77–6.71, *p *< 0.001), LVI (1.78, 1.03–3.1, *p *= 0.035), TNM stage (I and II vs. III: 2.78, 1.4–5.51, *p *= 0.003, I and II vs. IV: 7.75, 3.79–15.85, *p *< 0.001), chemotherapy (0.26, 0.14–0.47, *p *< 0.001), and SMG (1.79, 1.07–3.00, *p *= 0.025) were independently associated with OS (Tables 3 and 4).

### 3.6 Concordance rates of SMI, SMD, and SMG in the training and test sets

Using our definition of low SMG and previous cutoff values of low SMI and SMD, all three indicators, that is, low SMI, low SMD, and low SMG matched in only 18.7% (59/314) of patients in the training set and 16.2% (21/129) of patients in the test set among those with low values of SMI, SMD, or SMG (Figure S5). Except for the two patients in the training set, all patients (including the test set) with a low SMG were identified as patients belonging to either the low SMI or low SMD category.

### 3.7 Clinical utility of adding SMG to stage based on the C‐index comparison

The C‐index of stage (0.697, 95% CI = 0.657–0.735) showed superior discriminatory power compared with SMG (0.596, 95% CI = 0.559–0.633, estimated difference = 0.1, 95% CI = 0.045–0.159) in the training set (Table S2). When we compared a model using the stage with other model combining stage and SMG, the C‐index of the combined model (0.755, 95% CI = 0.719–0.79) showed superior discriminatory power compared with stage only (0.697, 95% CI = 0.657–0.735, estimated difference = 0.058, 95% CI = 0.027–0.089) (Table S3). These trends were maintained in the test set.

## 4 DISCUSSION

Our study demonstrated that SMG was an independent prognostic factor of OS, and the clinical impact of SMG might be comparable or better than that of skeletal muscle‐related parameters such as SMI and SMD in patients with CRC. However, the low concordance rate among SMI, SMD, and SMG in defining sarcopenic status might be a limitation in the clinical use of these indicators.

Over the decades, measurements of skeletal muscle have been investigated for prognostic or predictive value in different cancers including CRC.^4^, ^15^, ^17^, ^19^ Meta‐analyses demonstrated that SMI and SMD act as independent prognostic factors in terms of OS and cancer‐specific survival in various types of cancers.^8^, ^9^, ^10^ Recently, Weinberg and colleagues suggested the integration of SMI and SMD, and the novel integrated metric was defined as SMG.^13^ Shachar et al. investigated the clinical significance of SMG in patients with metastatic or early stage breast cancer and revealed that low SMG was associated with grade 3 or 4 toxicity and hospitalization in these patients.^11^, ^12^ Thus far, most studies on SMG have mainly focused on the prediction of adverse outcomes derived from chemotherapy.^11^, ^12^

It was reported that severe loss of SMG during staging surgery and adjuvant chemoradiotherapy was associated with worse OS in patients with endometrial cancer and that SMG could improve the prognostication in patients with advanced ovarian cancer.^14^, ^26^ Nevertheless, the clinical impact of SMG with respect to long‐term oncological outcomes has not been widely investigated, especially in patients with CRC. Previous studies showed that the combination of SMI and SMD could have a synergistic influence on the prediction of clinical outcomes in patients with CRC. Kroenke and colleagues revealed that the highest risk of mortality was observed in individuals with low SMD and sarcopenia (HR 2.02, 95% CI 1.65–2.47) compared to the low SMD or sarcopenia group as a result of using a combination of SMD and SMI.^16^ Similarly, Hopkins et al. demonstrated that patients with both low SMI and SMD were associated with poor survival outcomes with respect to OS, recurrence‐free survival, and cancer‐specific survival, whereas, myosteatosis and sarcopenia alone did not predict correctly all these survival outcomes.^19^ Xiao et al. also reported a stronger association in patients with concurrence of SMI and SMD with respect to major complications and mortality (HR 3.01, 95% CI 1.64–5.54 and HR 11.12, 95% CI 2.05–60.36, respectively) than in patients with SMI or SMD alone after analyzing 1630 colon cancer cases treated with surgery.^27^ Similarly, our study demonstrated that SMG showed a stronger prognostic impact than did SMI and SMD alone in the training and test sets, respectively.

With respect to risk stratification using SMG, the appropriate cutoff value has not been sufficiently investigated thus far. Shachar et al. reported that the optimal cutoffs for discriminating patients into low and high SMG categories were 1475 AU in the early stage of breast cancer (*n *= 151) and 1296 AU (as a median value) in metastatic breast cancer (*n *= 40) in two distinct studies.^11^, ^12^ Other studies defined cutoff values using the lowest tertile of SMG values.^14^, ^26^ These previous studies investigated the clinical significance of SMG in female patients with cancer; the studies had relatively small sample sizes; thus, comparison of the mean or median SMG values between the sexes has been scarcely reported. Hirai et al. reported that the mean SMG value was higher in men than in women (1729 AU vs. 1343 AU, *p *< 0.0001) who underwent surgeries for soft tissue sarcoma.^28^ Consistent with this result, our cohort showed a higher median SMG value in the male patients than in the female patients both in the training and test sets. Thus, we decided to define sex‐specific cutoff SMG values and confirmed that this newly developed stratification could be used as a significant prognostic factor even in an independent cohort. Nevertheless, the mean SMG value in our cohort (2454 AU in men and 1722 AU in women) seems to be higher than that reported previously, and the reason for this difference should be investigated in the future.

This study has several noteworthy aspects. We did not consider other anthropometric factors such as visceral or subcutaneous fat. Although some discordance was found in the impact of these adiposity‐related parameters on survival of patients with CRC,^6^, ^29^, ^30^ sarcopenic obesity has been regarded as a significant factor related to survival.^31^ In this respect, the correlation or interaction between SMG and adiposity might be an interesting issue. Although our study confirmed the clinical importance of the newly measured parameter in an independent cohort, it is not straightforward in that, our finding could be adopted in different regions showing diverse obesity phenotypes. Generally, obesity defined using BMI shows high diversity according to region or ethnicity.^32^ The synergistic effects of combining muscle density and mass must be validated in different ethnic groups. In our previous work, we found severe muscle loss within a 2‐ to 3‐month interval, although those patients had undergone preoperative chemoradiotherapy for locally advanced rectal cancer.^5^ Likewise, some concern was raised on the possible effects of the large interval between the pretreatment CT scan and the surgery date on the results; however, no concrete evidence was found. Although we included patients who had pretreatment scans obtained within 2 months, the median gap between CT and surgery was 9 days (IQR 6–15 days) in the training set and 8 days (IQR 4–15 days) in the test set. Finally, when the SMI, SMD, and SMG were all considered in determining the sarcopenic status in each patient, the rate of agreement of all three indicators was reported to be 18.7% (59/314) in the training set and 16.2% (21/129) in the test set, which might be quite low. This low concordance rate is likely to be a limiting point when making clinically important decisions based on skeletal muscle‐related indexes. Developing strategies to overcome this discordance according to each definition may be necessary, and this warrants further investigation.

In conclusion, our study shows that SMG was an independent significant factor for OS in patients with CRC. Combining SMI and SMD has a synergistic effect on improving prognostic accuracy as compared with SMI or SMD alone in the patients with CRC in our study. Our findings must be validated in different ethnicity groups.

## CONFLICT OF INTEREST

Nothing to declare.

## ETHICAL STATEMENTS

This retrospective study was conducted after approval by the institutional review boards (IRBs) of the hospitals, and the requirement for informed consent was waived in view of the retrospective design of the study.

## Supporting information

Supplementary Material

## Data Availability

The datasets generated and/or analyzed during the current study are available from the corresponding author upon reasonable request pending the approval of the institution(s) and trial/study investigators who contributed to the dataset.
