# Supplementary material for: Skeletal muscle gauge as a prognostic factor in patients with colorectal cancer
Source: Cancer Med. 2021 Oct 13;10(23):8451–61. doi: 10.1002/cam4.4354 (PMC8633260; doi:10.1002/cam4.4354)
Supplement: Supplementary file 1 — Supplementary Material [file CAM4-10-8451-s001.docx]

**Supplementary File**

**Figure S1. Inclusion of patients**

(A) Training set

**
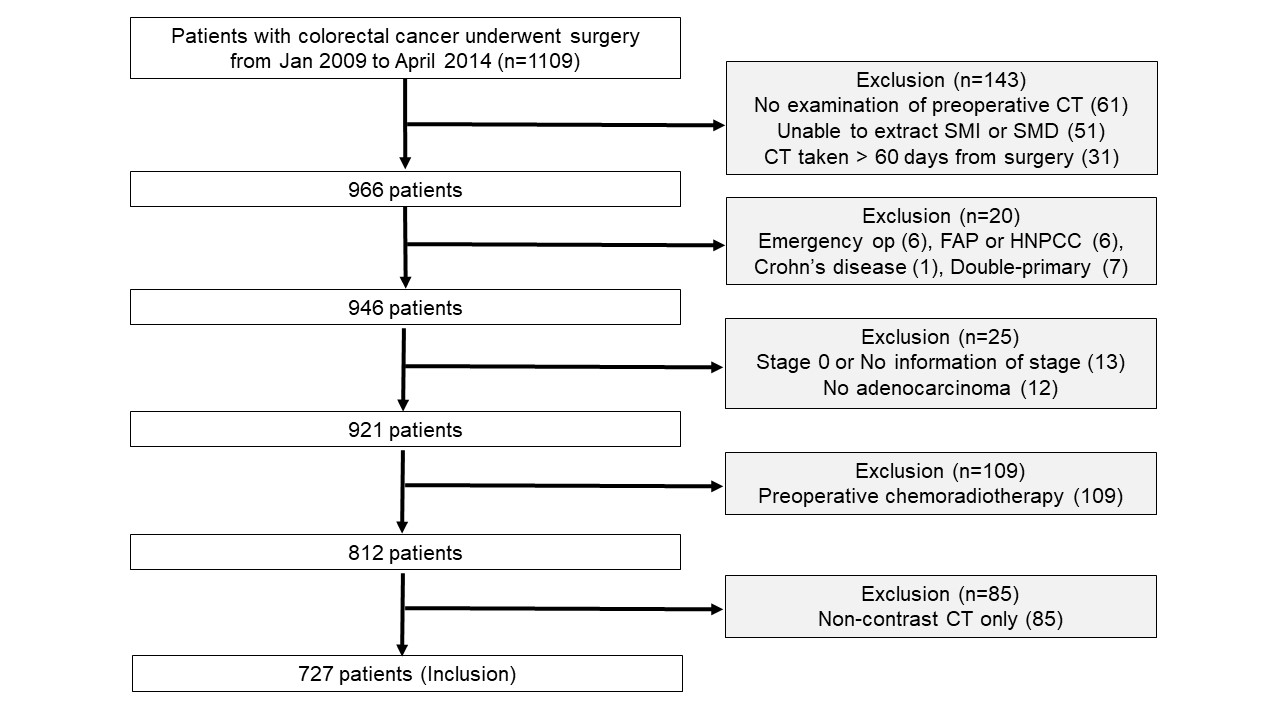
**

(B) Test set

**
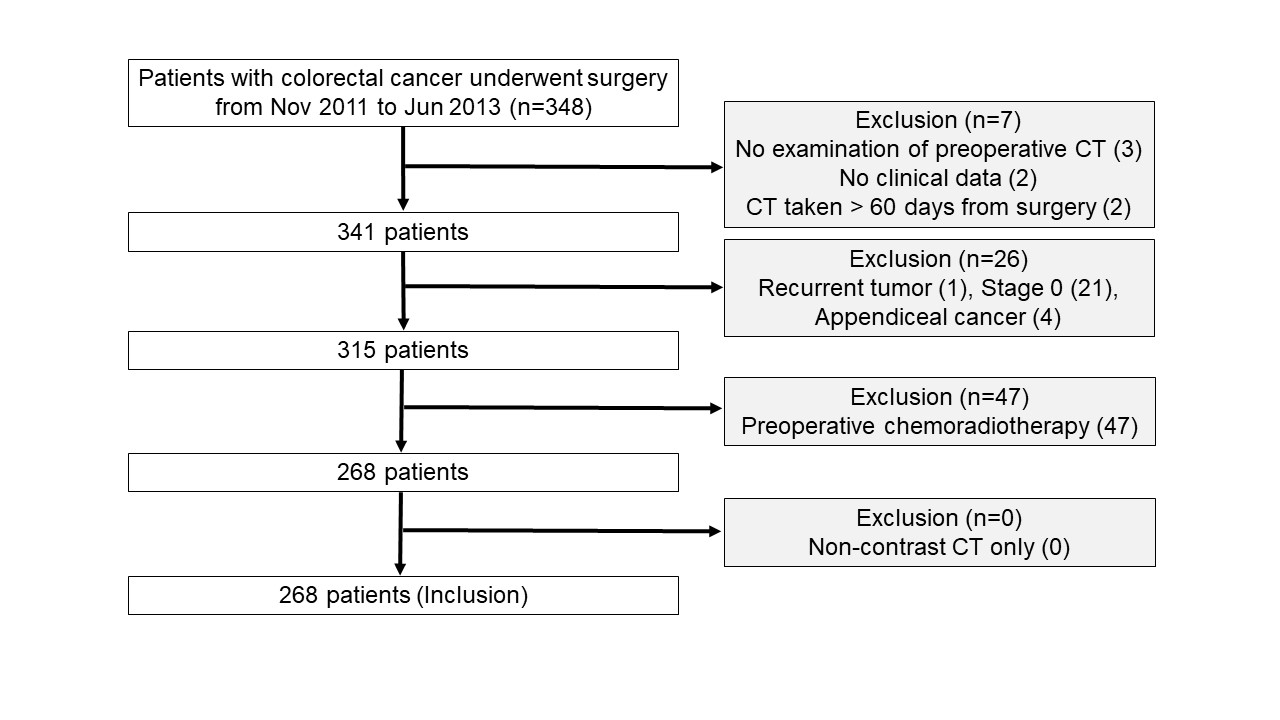
**

**Figure S2. Comparison of SMI, SMD and SMG according to sex in the training and test set**

| (A) Training set (n=727) |
| --- |
|  |
| 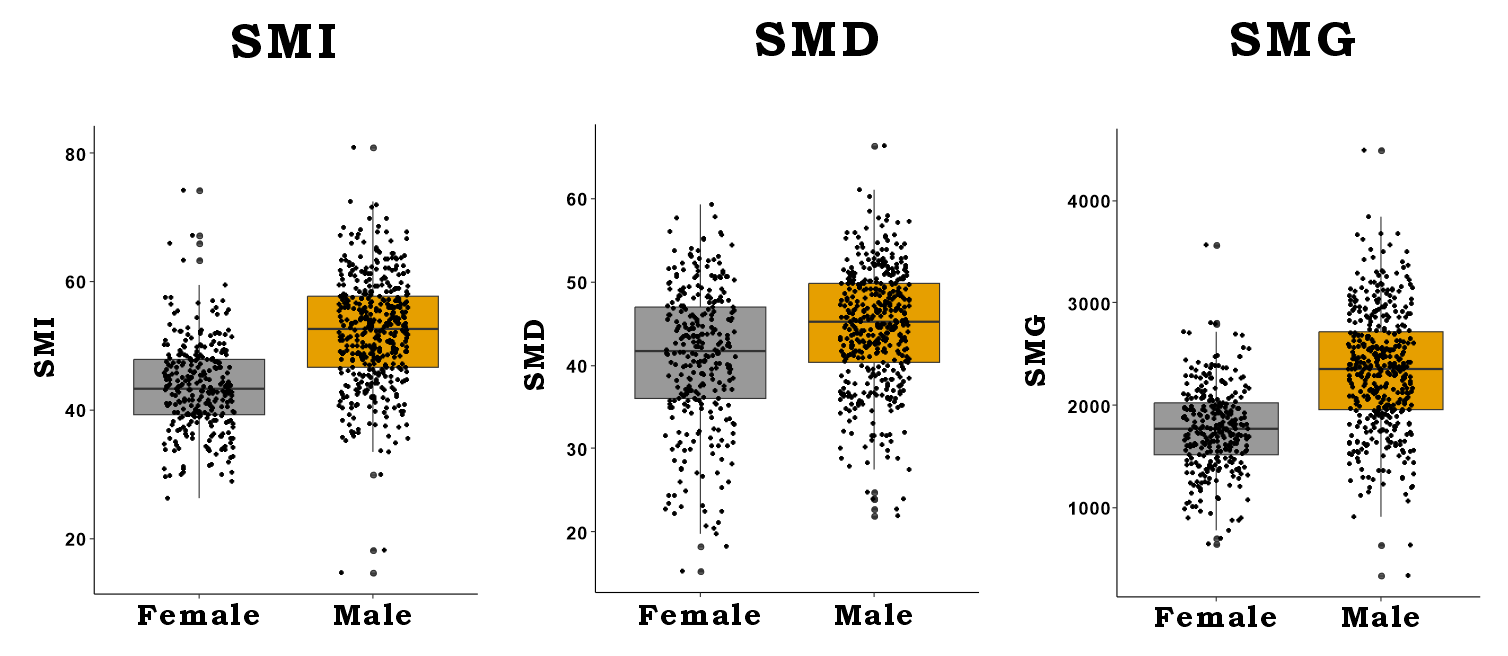 |
|  |
| (B) Test set (n=268) |
|  |
| 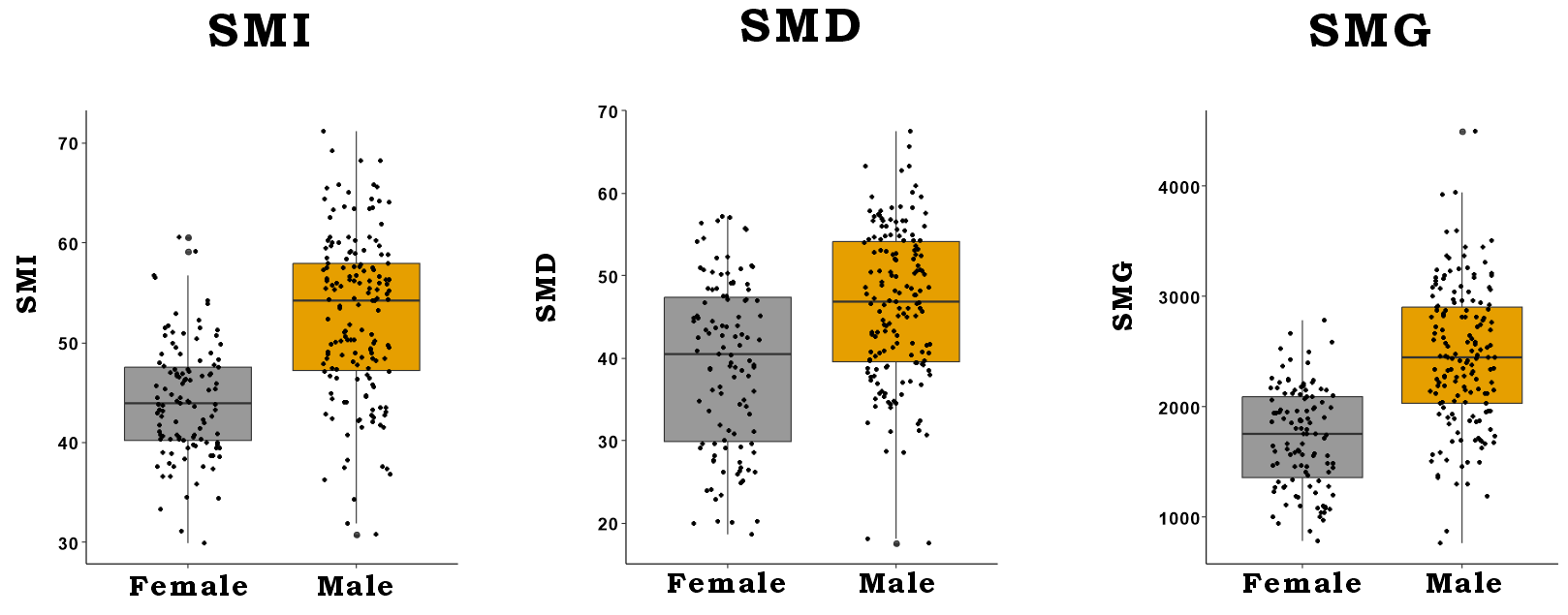 |

**Figure S3. Defining cut-off value of SMG according to the sex in the training set**

| (A) Survival analysis in male | (B) Histogram options in male |
| --- | --- |
| 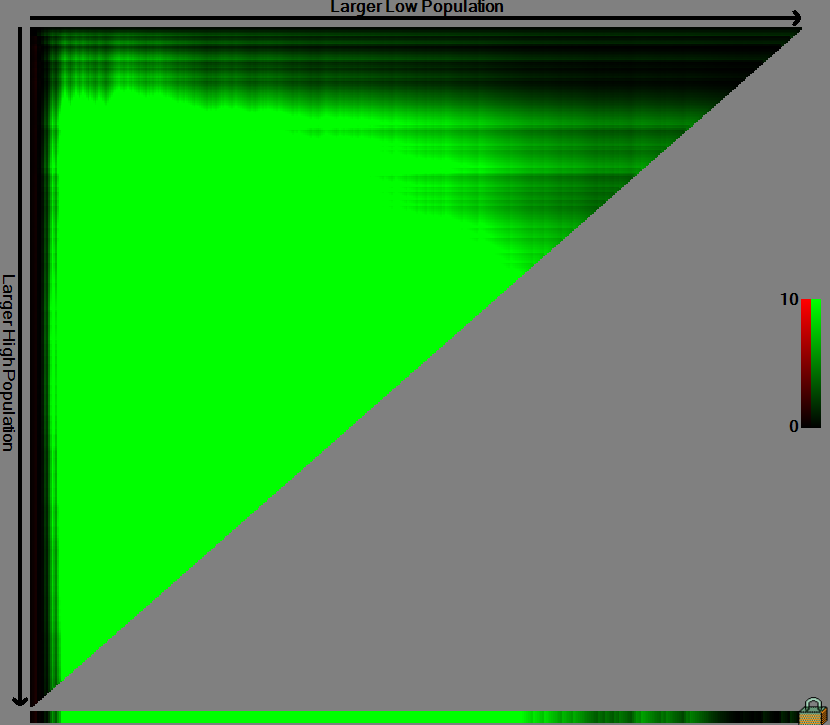 | 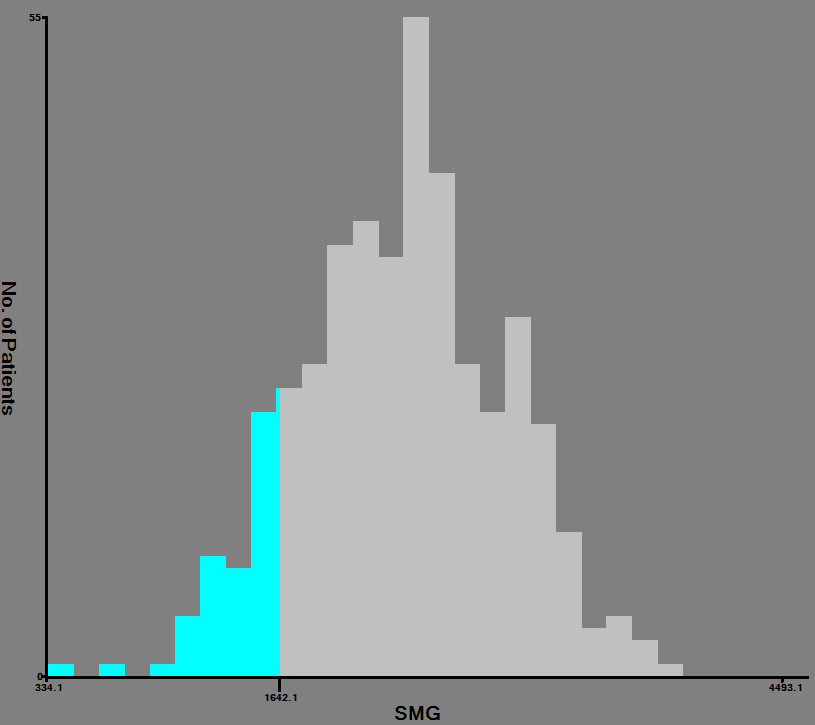 |
|  |  |
| (C) Survival analysis in female | (D) Histogram options in female |
| 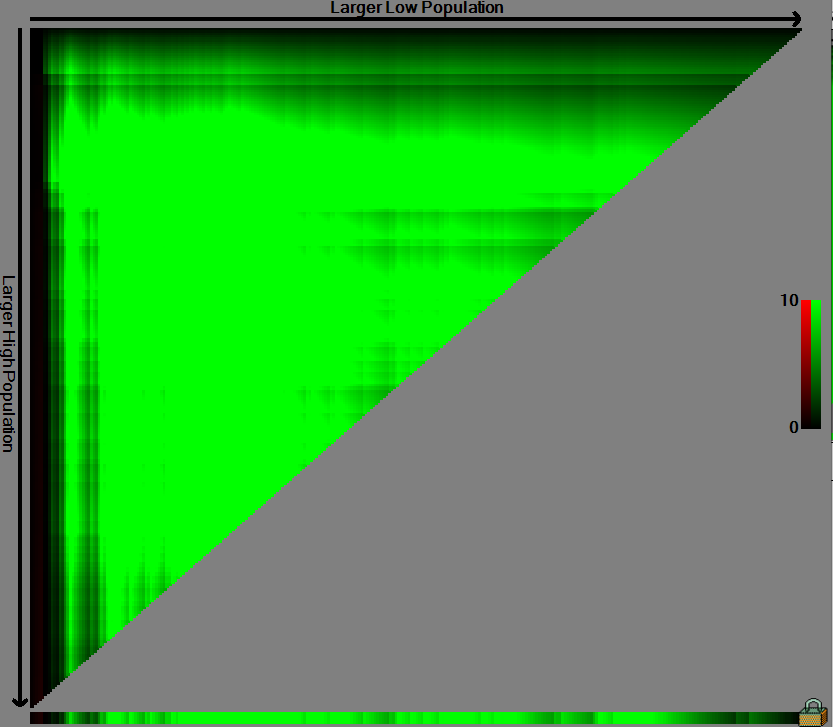 | 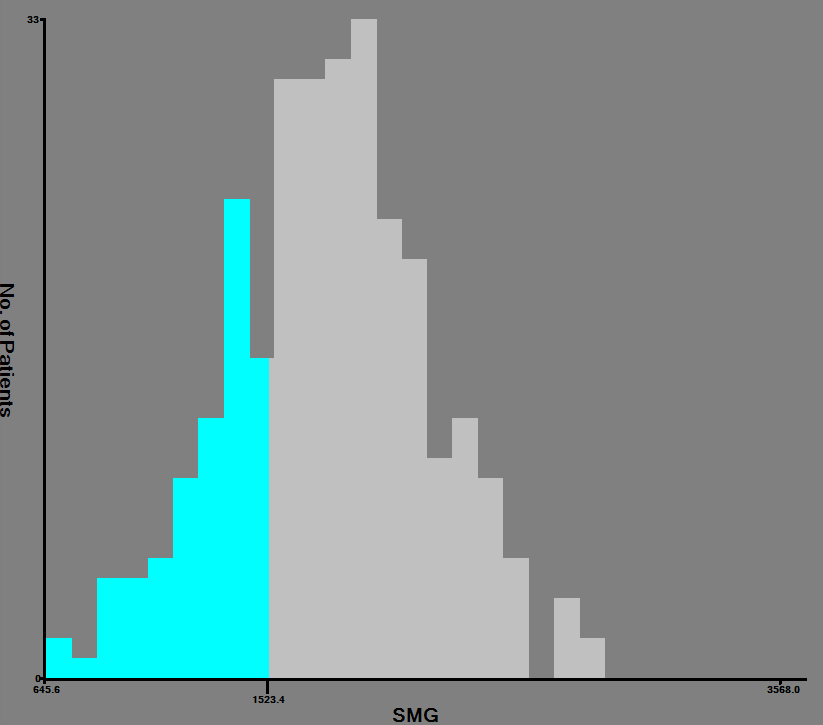 |

Figure legend: X-tile plots of the training set with respect to each sex are shown. The optimal cutoff point are shown on histograms as 1642.1 in male patients and 1523.4 in female patients respectively (B, D).

**Figure S4. Kaplan-Meier survival curve for OS according to SMI and SMD in the training and test set respectively**

| (A) SMI low vs. high in the training set | (B) SMD low vs. high in the training set |
| --- | --- |
| 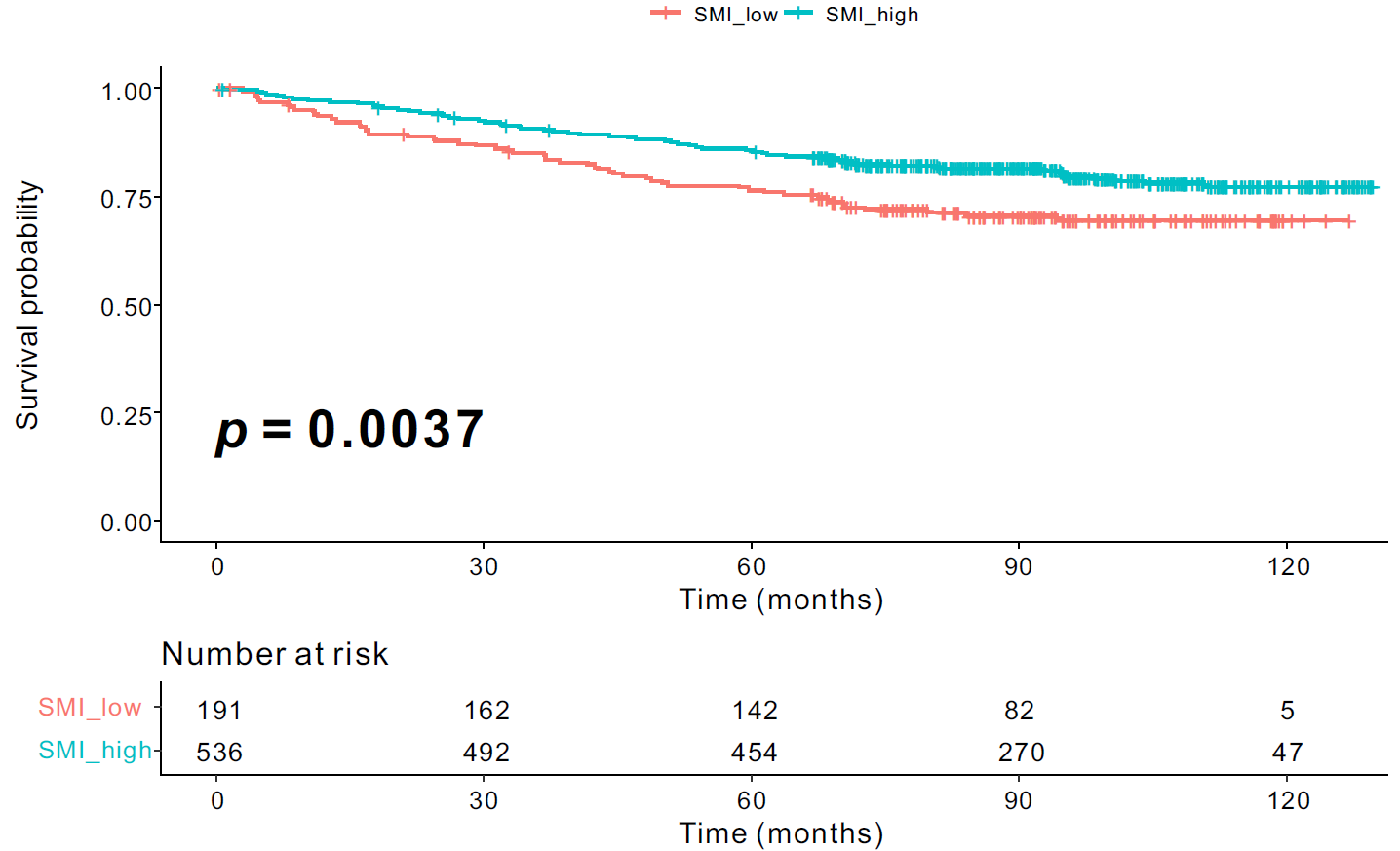 | 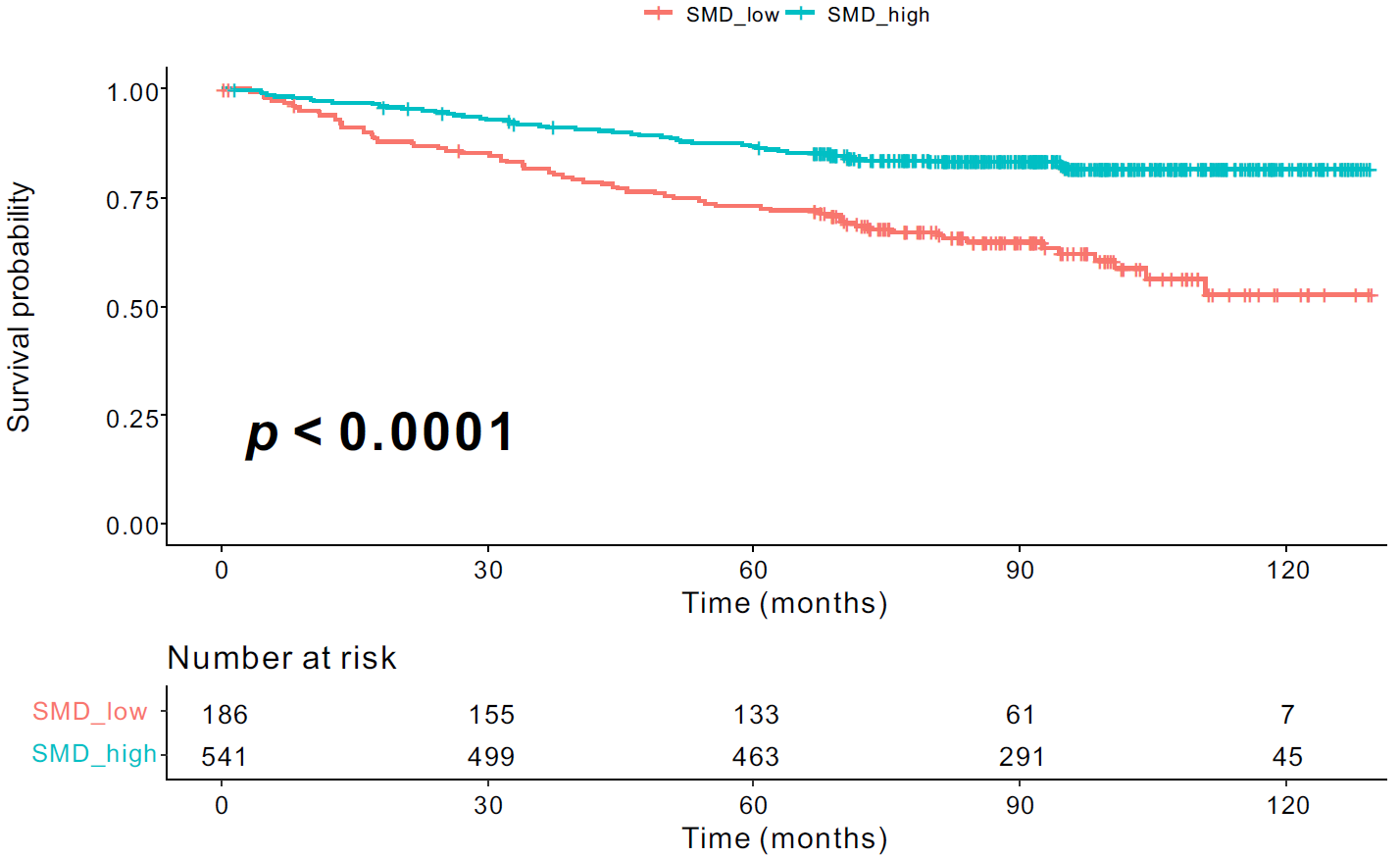 |
|  |  |
| (C) SMI low vs. high in the test set | (D) SMD low vs. high in the test set |
| 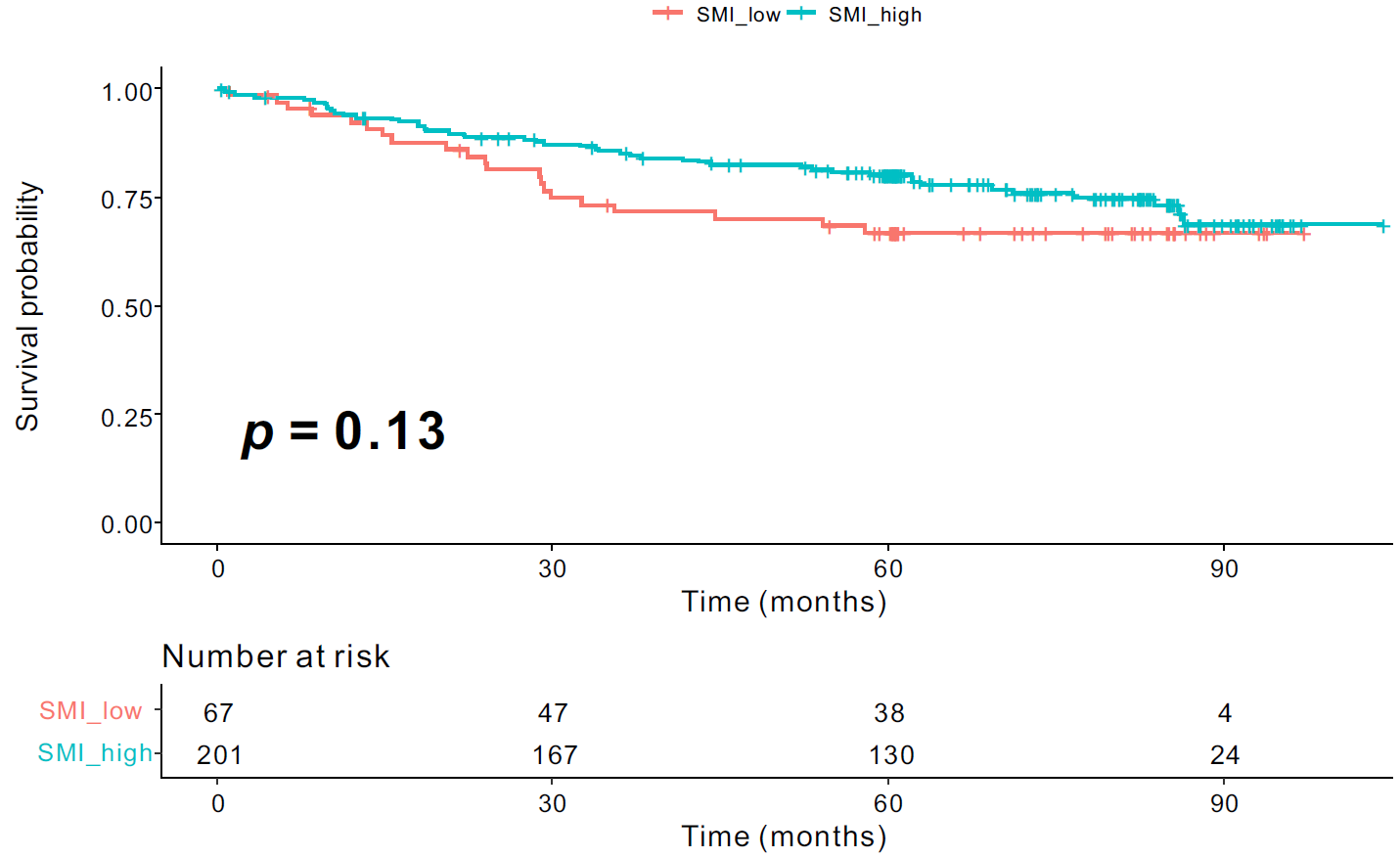 | 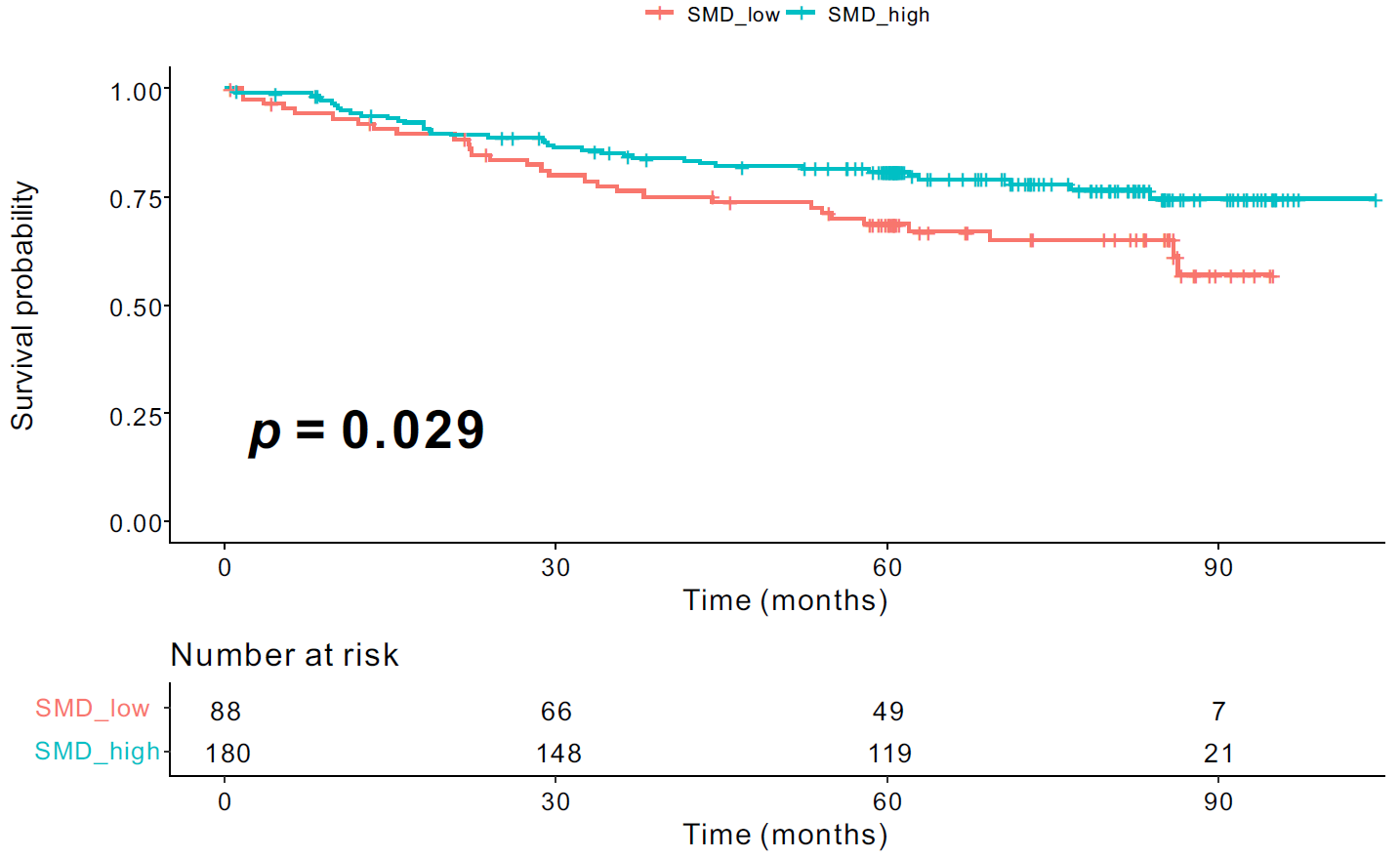 |

**Figure S5. Distribution of SMI, SMD and SMG in the training and test sets respectively**

| (A) Training set (n=727) | (B) Test set (n=268) |
| --- | --- |
| 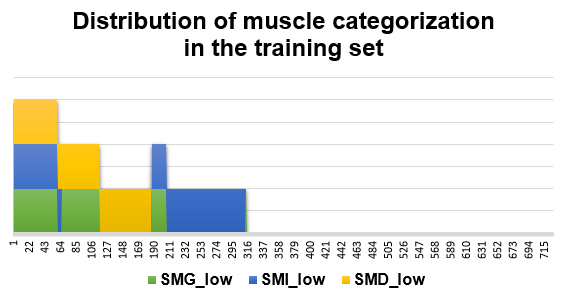 | 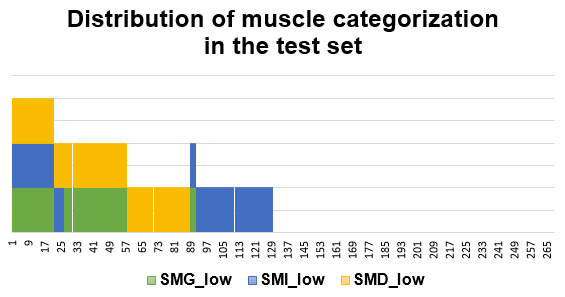 |

**Table S1. Cut-off values of SMI and SMD used in this study**

|  | SMI (cm^2^/m^2^) | SMD (HU) |
| --- | --- | --- |
| Reference | Martin et al. | |
| Male | 43 (BMI<25 kg/m^2^),  53 (BMI ≥25 kg/m^2^) | 41 (BMI<25 kg/m^2^),  33 (BMI ≥25 kg/m^2^) |
| Female | 41 | 41 (BMI<25 kg/m^2^),  33 (BMI ≥25 kg/m^2^) |

**Table S2. Comparison of C-index between stage versus SMG**

|  | Overall survival | | | |
| --- | --- | --- | --- | --- |
|  | Training set | | Test set | |
| Included variables | Stage | SMG | Stage | SMG |
| C-index (95% CI)  (bootstrapped) | 0.697  (0.657-0.735) | 0.596  (0.559-0.633) | 0.696  (0.632-0.756) | 0.592  (0.535-0.652) |
| Estimated difference | 0.1 (0.045-0.159) | | 0.104 (0.026-0.184) | |

C-index: Harrell's concordance index; CI: Confidence Interval

**Table S3. Comparison of C-index between stage versus stage plus SMG**

|  | Overall survival | | | |
| --- | --- | --- | --- | --- |
|  | Training set | | Test set | |
| Included variables | Stage + SMG | Stage | Stage + SMG | Stage |
| C-index (95% CI)  (bootstrapped) | 0.755  (0.719-0.79) | 0.697  (0.657-0.735) | 0.725  (0.656-0.787) | 0.696  (0.632-0.756) |
| Estimated difference | 0.058 (0.027-0.089) | | 0.029 (0.003-0.066) | |

C-index: Harrell's concordance index; CI: Confidence Interval
